# Osteoprotegerin and Cardiovascular Events in High‐Risk Populations: Meta‐Analysis of 19 Prospective Studies Involving 27 450 Participants

**DOI:** 10.1161/JAHA.118.009012

**Published:** 2018-08-15

**Authors:** Lena Tschiderer, Gerhard Klingenschmid, Rajini Nagrani, Johann Willeit, Jari A. Laukkanen, Georg Schett, Stefan Kiechl, Peter Willeit

**Affiliations:** ^1^ Department of Neurology Medical University of Innsbruck Austria; ^2^ Institute of Public Health and Clinical Nutrition University of Eastern Finland Kuopio Finland; ^3^ Central Finland Central Hospital Jyväskylä Finland; ^4^ Faculty of Sport and Health Sciences University of Jyväskylä Finland; ^5^ Department of Internal Medicine 3 University of Erlangen‐Nuremberg Erlangen Germany; ^6^ Department of Public Health and Primary Care University of Cambridge United Kingdom

**Keywords:** cardiovascular disease, high‐risk population, meta‐analysis, osteoprotegerin, prospective cohorts, Cardiovascular Disease, Biomarkers, Meta Analysis, Mortality/Survival

## Abstract

**Background:**

Osteoprotegerin is a cytokine involved in bone metabolism as well as vascular calcification and atherogenesis. Although circulating osteoprotegerin levels are robustly associated with incident cardiovascular disease (CVD) in the general population, its relevance as a biomarker among populations at high CVD risk is less clear.

**Methods and Results:**

Three independent reviewers systematically searched PubMed, EMBASE, and Web of Science to identify prospective studies that had recruited participants on the basis of having conditions related to high CVD risk. A total of 19 studies were eligible for inclusion, reporting on 27 450 patients with diabetes mellitus (2 studies), kidney disease (7 studies), preexisting heart disease (5 studies), or recent acute coronary syndromes (5 studies) at baseline. Over a mean follow‐up of 4.2 years, 4066 CVD events were recorded. In a random‐effects meta‐analysis, the pooled risk ratio for CVD events comparing people in the top versus the bottom tertile of osteoprotegerin concentration was 1.30 (95% confidence interval, 1.12–1.50; *P*<0.001; I^2^=68.3%). There was evidence for presence of publication bias (*P* value from Egger's test=0.013). Correction for publication bias using the trim‐and‐fill method reduced the risk ratio to 1.21 (95% confidence interval, 1.03–1.42; *P*<0.001). The risk ratios did not vary significantly by population type, geographical region, statistical adjustment, sample or assay type, age, sex, or length of follow‐up.

**Conclusions:**

In populations at high CVD risk, elevated circulating osteoprotegerin levels are associated with a higher risk for future CVD events. The magnitude of association appears weaker than in the general population.

**Clinical Perspective**

**What Is New?**

- In the present report, we systematically reviewed and combined the published evidence on the relevance of circulating osteoprotegerin concentration to cardiovascular events in high‐risk populations.
- Our meta‐analysis demonstrated a significant positive association between osteoprotegerin concentration and cardiovascular disease risk.
- The magnitude of association was similar in various clinically significant subgroups, including those defined by age and sex.

**What Are the Clinical Implications?**

- This work highlights the potential of osteoprotegerin as a biomarker for cardiovascular disease risk.

## Introduction

Osteoprotegerin is a member of the tumor necrosis factor (TNF) receptor superfamily and is involved in bone homeostasis.^1^ It inhibits osteoclastogenesis by binding to the receptor activator of nuclear factor‐κB ligand (RANKL), which prevents RANKL from binding to the receptor activator of nuclear factor‐κB (RANK).^2^ Inhibition of the RANK/RANKL pathway results in less osteoclast differentiation as well as reduced activation and survival of mature osteoclasts.^2^, ^3^ TNF‐related apoptosis‐inducing ligand, a protein that belongs to the TNF superfamily as well, also serves as an osteoprotegerin ligand.^4^ Osteoprotegerin thereby contributes to maintaining the balance between bone resorption and bone formation.^2^, ^5^

In addition to its role in bone homeostasis, osteoprotegerin has been implicated in the development of cardiovascular diseases (CVDs).^6^ It is found in atherosclerotic plaques,^7^, ^8^ may regulate vascular calcification,^9^, ^10^ and may thereby influence cardiovascular risk. Furthermore, genetic studies have demonstrated associations of osteoprotegerin gene polymorphisms with CVD.^11^, ^12^, ^13^, ^14^, ^15^ In a literature‐based meta‐analysis of 9 general population studies, we recently demonstrated robust positive associations between osteoprotegerin concentration and incident CVD.^16^ However, it is unclear whether these associations equally apply to high‐risk populations. Although several individual studies have investigated the predictive significance of osteoprotegerin in these settings,^17^, ^18^, ^19^, ^20^, ^21^, ^22^, ^23^, ^24^, ^25^, ^26^, ^27^, ^28^, ^29^, ^30^, ^31^, ^32^, ^33^, ^34^, ^35^ the interpretation of their findings has been complicated by differing in scales of association, levels of adjustment, and outcome definitions.

The principal aim of this report is to review comprehensively the available literature and to perform a meta‐analysis of reported associations between osteoprotegerin and risk for future CVD in high‐risk populations (ie, in studies that have recruited participants on the basis of having conditions related to high CVD risk). Secondary analyses will assess associations with coronary heart disease (CHD) events and stroke separately and will clarify whether the magnitude of association differs according to study‐level characteristics.

## Methods

### Research Data Availability

The database of published results from studies included in the meta‐analysis is made available to other researchers for purposes of reproducing the results or replicating the procedure.^36^

### Literature Search, Study Selection, and Data Extraction

We systematically sought PubMed, Web of Science, and EMBASE for prospective studies published between January 1970 and April 2017 that reported on associations of osteoprotegerin concentration with CVD outcomes (defined as nonfatal CHD [ie, myocardial infarction, unstable or stable angina, or coronary revascularization procedures], nonfatal stroke, or cardiovascular death). We also scanned reference lists of articles (including reviews) and corresponded with several study investigators. Table S1 provides a detailed description of search terms used in the literature search. Studies were eligible for inclusion if they (1) had a prospective design; (2) had recruited study participants on the basis of having preexisting conditions favoring risk of future CVD; and (3) had recorded incident CVD outcomes over a period of >1 month. Studies that did not report on the predefined outcome definition (including those reporting on the combination of CVD events and all‐cause mortality) were excluded from the analysis.

For each eligible study, 3 reviewers (L.T., G.K., P.W.) independently extracted the following pieces of information: type of baseline disease, study location, year of baseline, duration of follow‐up, mean or median age at baseline, proportion of male participants, osteoprotegerin assay type (ELISAs versus immunofluorometric assays), osteoprotegerin assay manufacturers, and sample types (plasma versus serum). In addition, we extracted information on the statistical adjustment used, categorizing adjustments as “unadjusted” if no adjustment was employed; “+” for adjustment for age and sex; “++” for adjustment for age, sex, and non–blood‐based risk factors, such as smoking, blood pressure, and diabetes mellitus; and “+++” for further adjustments for blood‐based risk factors (eg, cholesterol and C‐reactive protein). If a study reported different adjustment models, the most adjusted model was used to minimize the scope for confounding. If information about the same study was published twice or more often, we used the most recent publication. Study quality was evaluated using the Newcastle‐Ottawa scale for cohort studies.^37^ The meta‐analysis was performed following the Preferred Reporting Items for Systematic Reviews and Meta‐Analyses (PRISMA) guidelines.^38^

### Statistical Analyses

We conducted analyses according to a predefined statistical analysis plan. The primary outcome was CVD events (as defined above); secondary outcomes were CHD events and stroke events. Because studies reported effect estimates on different scales (eg, per standard deviation or across quartiles), we converted risk ratios and 95% confidence intervals (CIs) to reflect a comparison of the top versus bottom tertiles of baseline osteoprotegerin distribution using methods described elsewhere.^39^ One study^29^ did not provide sufficient information on the osteoprotegerin distribution—a prerequisite for converting its risk ratio—and we therefore estimated its distribution on the basis of comparable study populations.^26^, ^28^, ^30^ We pooled study‐specific risk ratios using random‐effects meta‐analysis (sensitivity analyses used fixed‐effect meta‐analysis). The I^2^ statistic was used to assess heterogeneity across studies.^40^ Subgroup analyses were conducted using meta‐regression across prespecified study‐level characteristics.^40^ We evaluated whether publication bias was present by visually inspecting a funnel plot and applying Egger's asymmetry test.^41^ We estimated a risk ratio corrected for publication bias using the trim‐and‐fill method, which imputes artificial studies to achieve symmetry of the funnel plot.^42^ In addition, to evaluate the influence of single studies on the overall result, we performed a leave‐one‐out cross‐validation, which reestimates the pooled risk ratio while omitting each study in turn. All statistical tests were 2‐sided; *P*<0.05 was deemed as statistically significant. Data were analyzed using the statistical software Stata, version 14.1 (StataCorp). Because our analysis relied entirely on data available in the published literature, approval by the institutional review board of the project was not required.

## Results

### General Characteristics of Included Studies

**Figure 1:**
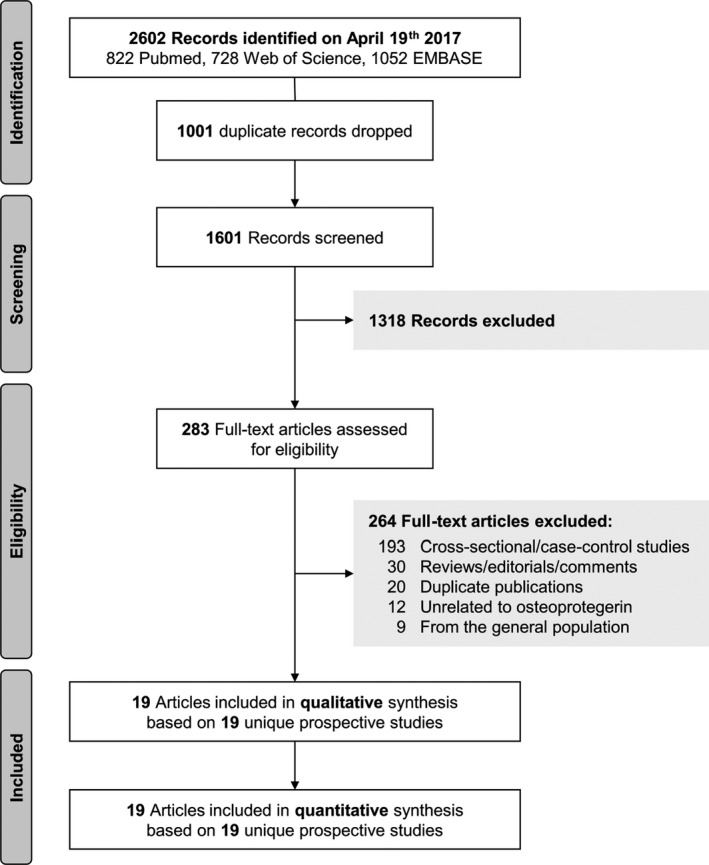
Study flow diagram.

Of 2602 records retrieved from PubMed, Web of Science, and EMBASE, we excluded 1001 duplicates and 1318 records after review of titles and abstracts (Figure 1). When reviewing the full text of the remaining 283 articles, we excluded a further 264 additional articles, leaving 19 prospective studies^17^, ^18^, ^19^, ^20^, ^21^, ^22^, ^23^, ^24^, ^25^, ^26^, ^27^, ^28^, ^29^, ^30^, ^31^, ^32^, ^33^, ^34^, ^35^ eligible for inclusion in the meta‐analysis. Patients were recruited on the basis of having diabetes mellitus in 2 studies, kidney disease in 7 studies, preexisting heart disease in 5 studies, and recent acute coronary syndromes in 5 studies. Details on the definitions of baseline conditions are provided in Table S2. Twelve studies were based in Europe, 4 were based in Asia, and 3 were located in multiple continents (Table 1). Of the 19 prospective studies, 7 were nested in a trial. The weighted mean age was 60.9 years; 68.3% of patients were men. The average quality of the studies assessed by the Newcastle‐Ottawa scale for cohort studies was 7.6. For measuring osteoprotegerin concentrations, 15 studies used ELISAs and 4 studies used immunofluorometric assays. Ten studies had measured osteoprotegerin concentration in plasma, and 9 had measured osteoprotegerin concentration in serum. In total, the studies involved 27 450 participants and reported on 4066 CVD outcomes recorded over a weighted mean follow‐up duration of 4.2 years (Table 2). One study reported unadjusted effect estimates; another 3 studies reported effect estimates adjusted for age, sex, and non–blood‐based markers; 14 studies reported multivariable adjusted effect estimates (including blood‐based markers); and 1 study reported unadjusted risk ratios for CVD events and multivariable adjusted risk ratios for stroke.

**Table 1:** Design Features of Contributing Studies

Study Acronym or First Author	Location	Year of Baseline, Range	Study Quality, NOS	Mean Age, y	Male Sex, %	Osteoprotegerin Assay Type (Manufacturer)	Sample Type
Populations with diabetes mellitus at baseline							
Anand^17^	United Kingdom	NR	7	52.7	60.6	ELISA (Biomedica)	Plasma
FINNDIANE^18^	Finland	1997–2004	8	36.9	49.8	IFMA (R&D Systems)	Serum
Populations with kidney disease at baseline							
ALERT^19^, ^a^	Multicenter	1997	7	49.6	65.8	ELISA (Biomedica)	Serum
CRISIS^20^	United Kingdom	2002–2010	6	63.8	61.8	ELISA (BioVendor)	Plasma
Kuzniewski^21^	Poland	2004	8	60.0	56.5	ELISA (BioVendor)	Plasma
Nakashima^22^	Japan	2003	7	62.1	56.3	ELISA (Immundiagnostik)	Plasma
Nishiura^23^	Japan	2000–2006	7	58.9	65.7	ELISA (Immundiagnostik)	Serum
Speer^24^	Hungary	2004–2007	7	63.4	61.2	ELISA (Immundiagnostik)	Serum
Yilmaz^25^	Turkey	2009–2013	7	48.9	51.9	ELISA (RayBiotech)	Serum
Populations with preexisting heart disease at baseline							
CLARICOR^26^, ^a^	Denmark	1999–2000	9	65.4	69.4	IFMA (R&D Systems)	Serum
CORONA^27^, ^a^	The Netherlands	2003–2005	8	72.0	76.7	ELISA (R&D Systems)	Plasma
Jono^28^	Japan	1999–2000	8	63.1	82.7	ELISA (Cosmo Bio)	Serum
PEACE^29^, ^a^	Multicenter	1996–2000	6	63.7	81.0	ELISA (R&D Systems)	Plasma
Pedersen (1)^30^	Norway	2000–2001	9	62.0^b^	71.9	ELISA (R&D Systems)	Serum
Populations with recent acute coronary syndromes at baseline							
MERLIN‐TIMI36^31^, ^a^	Italy	2004–2006	8	64.0	64.9	IFMA (R&D Systems)	Plasma
OPTIMAAL^32^	Multicenter	1998–1999	9	67.8	70.3	ELISA (R&D Systems)	Plasma
PLATO^33^, ^a^	Multicenter	2006–2008	6	62.0	71.6	ELISA (NR)	Plasma
PRACSIS^34^	Sweden	1996–2001	9	65.0	70.7	ELISA (R&D Systems)	Serum
Pedersen (2)^35^	Denmark	2006–2008	9	63.5	41.3	IFMA (R&D Systems)	Plasma
Total		1996–2013	7.6	60.9	68.3		

Summary statistics are ranges, weighted means, or sums, as appropriate. ALERT indicates Assessment of Lescol in Renal Transplantation Study; CLARICOR, Effect of Clarithromycin on Mortality and Morbidity in Patients With Ischemic Heart Disease; CORONA, Controlled Rosuvastatin Multinational Trial; CRISIS, Chronic Renal Insufficiency Standards Implementation Study; FINNDIANE, Finnish Diabetic Nephropathy Study; IFMA, immunofluorometric assay; MERLIN‐TIMI36, Metabolic Efficiency With Ranolazine for Less Ischemia in Non–ST‐Elevation Acute Coronary Syndromes Trial; NOS, Newcastle‐Ottawa scale; NR, not reported; OPTIMAAL, Optimal Trial in Myocardial Infarction With Angiotensin II Antagonist Losartan; PEACE, Prevention of Events With Angiotensin Converting Enzyme Inhibition Trial; PLATO, Platelet Inhibition and Patient Outcomes Trial; PRACSIS, Prognosis and Risk in Acute Coronary Syndrome in Sweden.

a Nested in clinical trial.

b Median.

**Table 2:** Follow‐Up Data in the Contributing Studies

Study Acronym or First Author	Maximum Follow‐Up, y	No. of Participants	No. of Events			Adjustment of Reported Risk Ratio
			CVD	CHD	Stroke	
Populations with diabetes mellitus at baseline						
Anand^17^	1.5^a^	510	16	···	···	Unadjusted
FINNDIANE^18^	10.5^a^	1903	190	152	71	++
Populations with kidney disease at baseline						
ALERT^19^, ^b^	6.7^a^	1889	···	285	···	+++
CRISIS^20^	3.8^a^	463	108	···	···	+++
Kuzniewski^21^	7.0	69	31	···	···	+++
Nakashima^22^	6.0	151	40	···	···	++
Nishiura^23^	3.5^a^	99	27	···	···	+++
Speer^24^	2.6	98	23	···	···	+++
Yilmaz^25^	3.0^d^	291	87	···	···	+++
Populations with preexisting heart disease at baseline						
CLARICOR^26^, ^b^	2.6^a^	4063	623	303	146	+++
CORONA^27^, ^b^	3.0	1464	318	255	···	+++
Jono^28^	5.1^a^	225	101	···	···	+++
PEACE^29^, ^b^	7.0	3767	1290	···	NR	Unadjusted/+++^c^
Pedersen (1)^30^	6.1^d^	1025	60	103	···	+++
Populations with recent acute coronary syndromes at baseline						
MERLIN‐TIMI36^31^, ^b^	0.9^d^	4463	544	336	···	+++
OPTIMAAL^32^	2.3^a^	234	26	···	···	+++
PLATO^33^, ^b^	2.4	5123	432	···	···	++
PRACSIS^34^	10.1	897	150	107	43	+++
Pedersen (2)^35^	2.3^d^	716	···	51	···	+++
Total	4.2	27 450	4066	1592	260	

Summary statistics are weighted means or sums, as appropriate. ++ indicates adjusted for age, sex, and non–blood‐based risk factors; +++, further adjusted for blood‐based risk factors; ALERT, Assessment of Lescol in Renal Transplantation Study; CHD, coronary heart disease; CRISIS, Chronic Renal Insufficiency Standards Implementation Study; CVD, cardiovascular disease; FINNDIANE, Finnish Diabetic Nephropathy Study; MERLIN‐TIMI36, Metabolic Efficiency With Ranolazine for Less Ischemia in Non–ST‐Elevation Acute Coronary Syndromes Trial; NR, study did not report the number of stroke events (despite reporting hazard ratios for stroke); OPTIMAAL, Optimal Trial in Myocardial Infarction With Angiotensin II Antagonist Losartan; PLATO, Platelet Inhibition and Patient Outcomes Trial; PRACSIS, Prognosis and Risk in Acute Coronary Syndrome in Sweden.

a Mean.

b Nested in clinical trial.

c Median.

d Study reported unadjusted risk ratios for the outcome cardiovascular events and multiple adjustment for the outcome stroke.

### Overall Association of Osteoprotegerin With Cardiovascular Events

**Figure 2:**
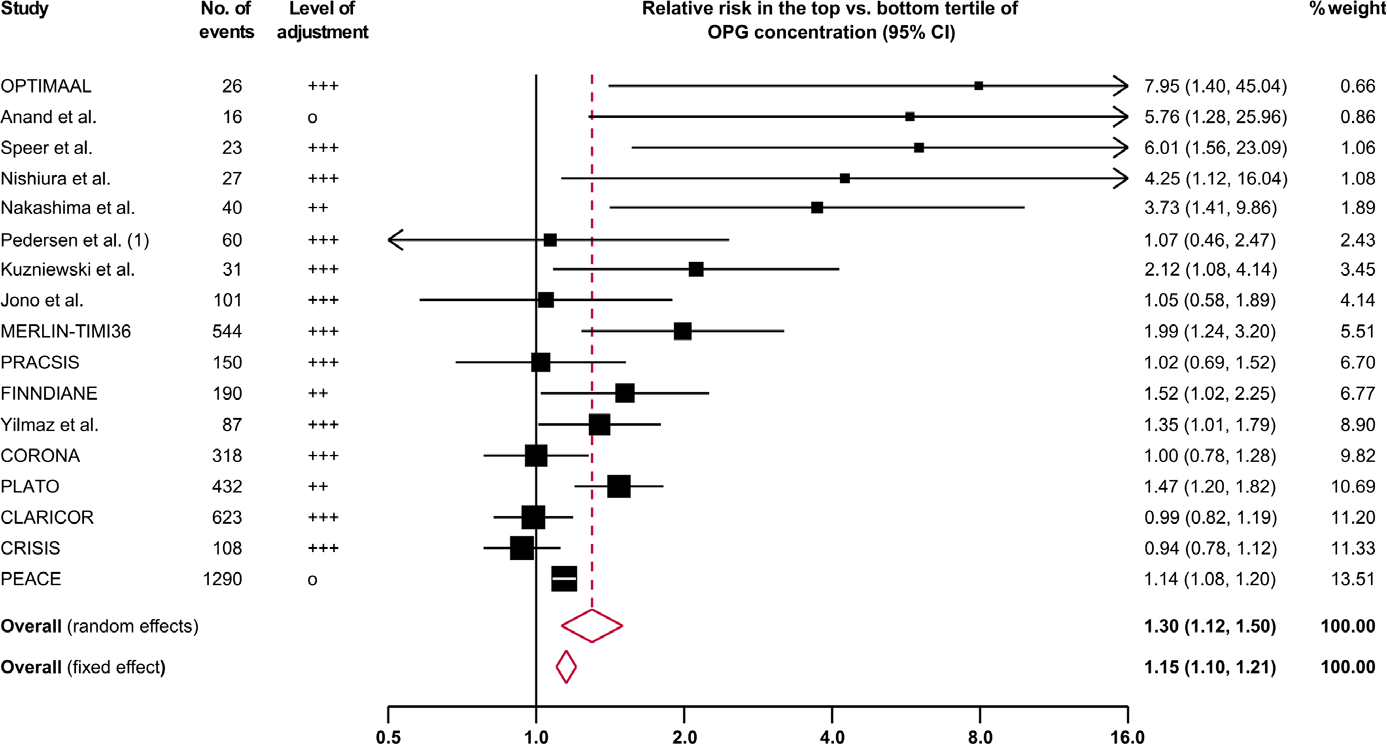
Combined relative risk for cardiovascular events in the top vs the bottom tertile of osteoprotegerin (OPG) concentration. Sizes of data markers indicate the weight of each study in the analysis. The I^2^ value was 68.3% (*P*<0.001). CI indicates confidence interval; CLARICOR, Effect of Clarithromycin on Mortality and Morbidity in Patients With Ischemic Heart Disease; CORONA, Controlled Rosuvastatin Multinational Trial; CRISIS, Chronic Renal Insufficiency Standards Implementation Study; FINNDIANE, Finnish Diabetic Nephropathy Study; MERLIN‐TIMI36, Metabolic Efficiency With Ranolazine for Less Ischemia in Non–ST‐Elevation Acute Coronary Syndromes Trial; OPTIMAAL, Optimal Trial in Myocardial Infarction With Angiotensin II Antagonist Losartan; PEACE, Prevention of Events With Angiotensin Converting Enzyme Inhibition Trial; PLATO, Platelet Inhibition and Patient Outcomes Trial; PRACSIS, Prognosis and Risk in Acute Coronary Syndrome in Sweden.

Figure 2 shows the forest plot of the association of baseline osteoprotegerin concentration with incident CVD events. The pooled relative risk for CVD events was 1.30 (95% CI, 1.12–1.50; *P*<0.001) for a comparison of individuals in the top versus the bottom tertile of baseline osteoprotegerin concentration. There was a high degree of between‐study heterogeneity (I^2^=68.3%; *P*<0.001). In comparison, a fixed‐effect meta‐analysis yielded a pooled risk ratio of 1.15 (95% CI, 1.10–1.21; *P*<0.001).

**Figure 3:**
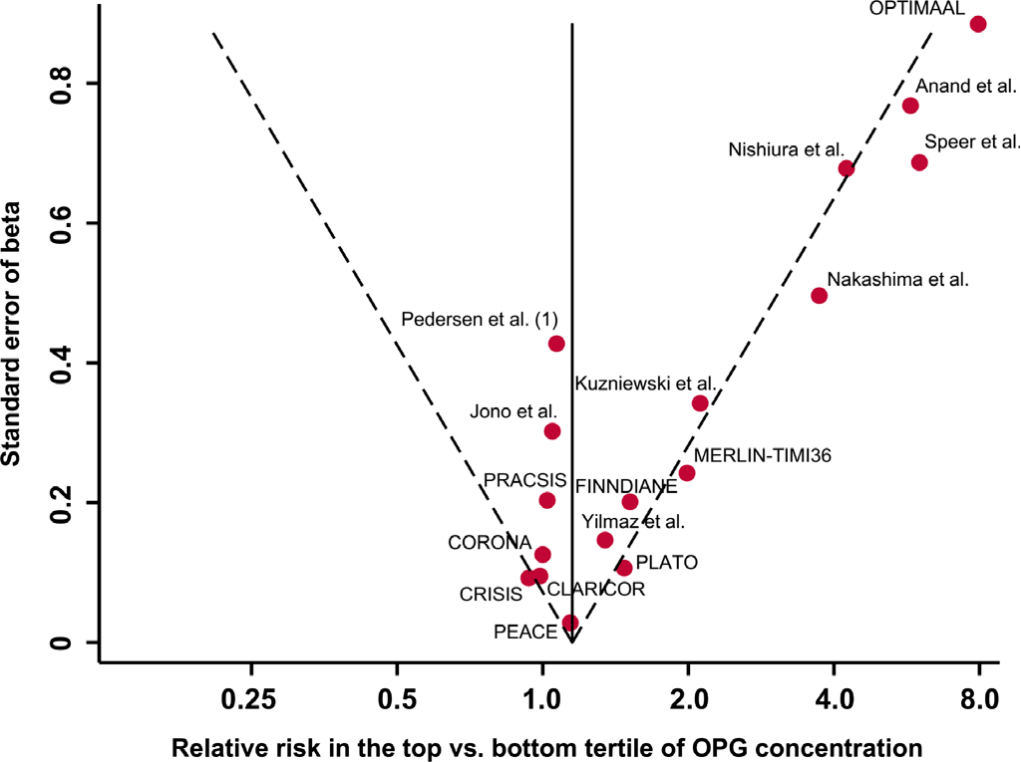
Funnel plot of reported associations between osteoprotegerin (OPG) concentration and risk of cardiovascular events. The dotted lines show pseudo 95% confidence intervals around the overall pooled estimate. The *P* value from Egger's asymmetry test of associations was 0.013. CLARICOR indicates Effect of Clarithromycin on Mortality and Morbidity in Patients With Ischemic Heart Disease; CORONA, Controlled Rosuvastatin Multinational Trial; CRISIS, Chronic Renal Insufficiency Standards Implementation Study; FINNDIANE, Finnish Diabetic Nephropathy Study; MERLIN‐TIMI36, Metabolic Efficiency With Ranolazine for Less Ischemia in Non–ST‐Elevation Acute Coronary Syndromes Trial; OPTIMAAL, Optimal Trial in Myocardial Infarction With Angiotensin II Antagonist Losartan; PEACE, Prevention of Events With Angiotensin Converting Enzyme Inhibition Trial; PLATO, Platelet Inhibition and Patient Outcomes Trial; PRACSIS, Prognosis and Risk in Acute Coronary Syndrome in Sweden.

**Figure 4:**
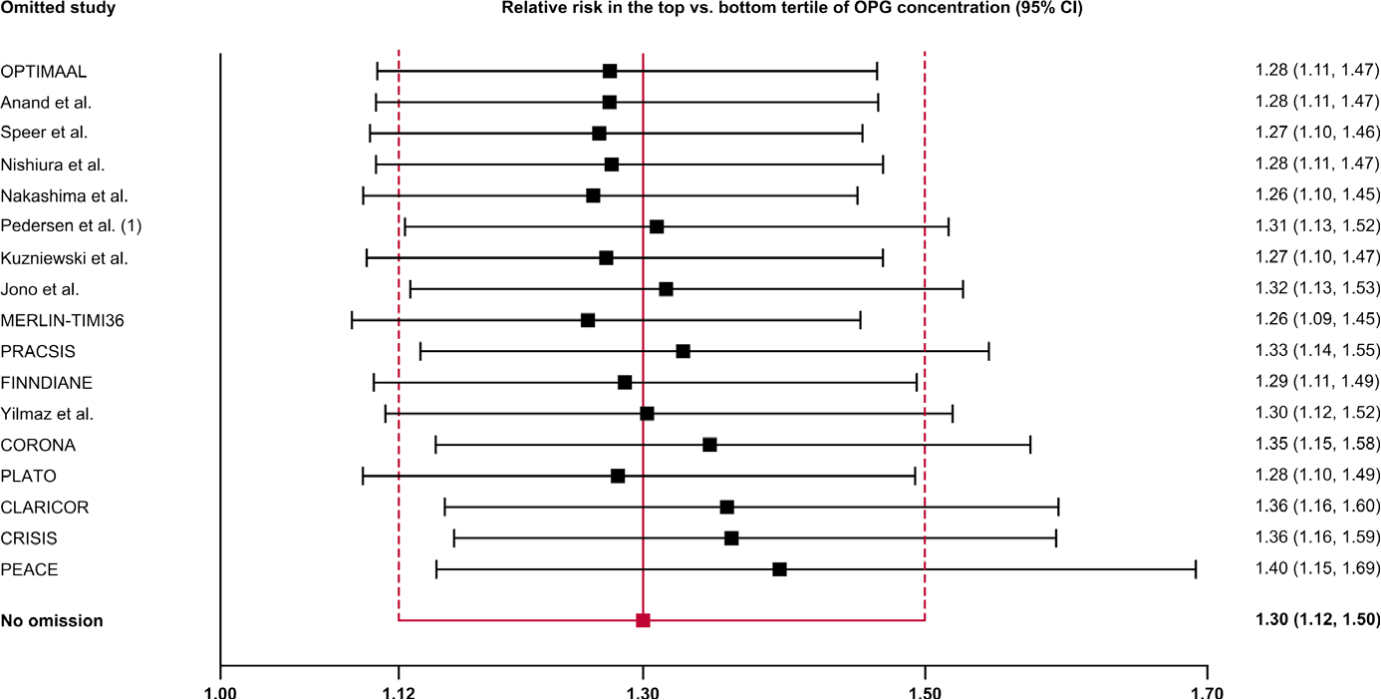
Reestimated pooled risk ratios for cardiovascular outcomes omitting one study in each turn. CI indicates confidence interval; CLARICOR, Effect of Clarithromycin on Mortality and Morbidity in Patients With Ischemic Heart Disease; CORONA, Controlled Rosuvastatin Multinational Trial; CRISIS, Chronic Renal Insufficiency Standards Implementation Study; FINNDIANE, Finnish Diabetic Nephropathy Study; MERLIN‐TIMI36, Metabolic Efficiency With Ranolazine for Less Ischemia in Non–ST‐Elevation Acute Coronary Syndromes Trial; OPG, osteoprotegerin; OPTIMAAL, Optimal Trial in Myocardial Infarction With Angiotensin II Antagonist Losartan; PEACE, Prevention of Events With Angiotensin Converting Enzyme Inhibition Trial; PLATO, Platelet Inhibition and Patient Outcomes Trial; PRACSIS, Prognosis and Risk in Acute Coronary Syndrome in Sweden.

There was evidence for publication bias, as indicated by the funnel plot (Figure 3) and a significant Egger's asymmetry test (*P*=0.013). Using the trim‐and‐fill method, 5 additional artificial studies were included into the meta‐analysis to generate a symmetric funnel plot (Figure S1). This correction for publication bias yielded a relative risk of 1.21 (95% CI, 1.03–1.42; *P*=0.020). Reestimated pooled risk ratios by omitting each study in turn remained significant for all omissions (Figure 4).

**Figure 5:**
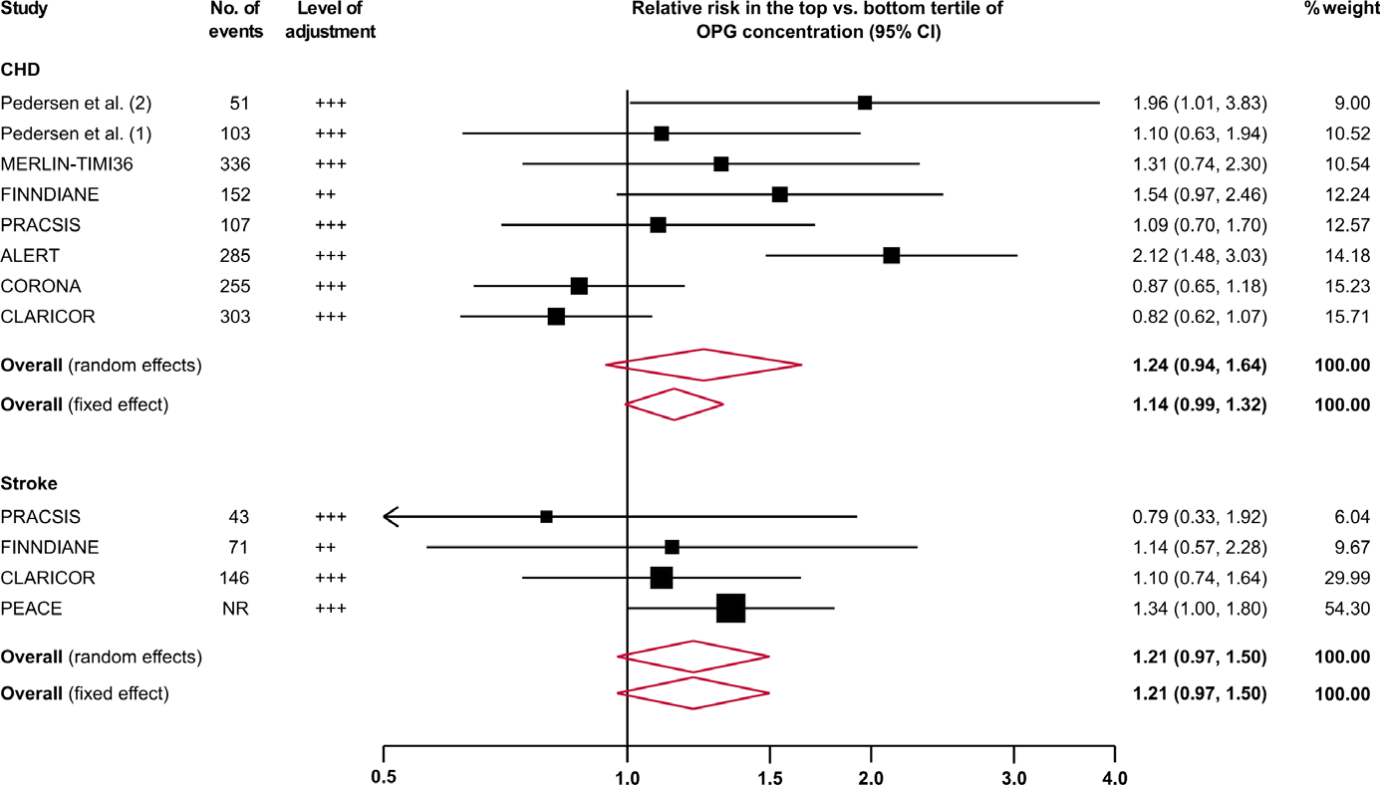
Combined relative risk for future coronary heart disease (CHD) and stroke in the top vs the bottom tertile of osteoprotegerin (OPG) concentration. Sizes of data markers indicate the weight of each study in the analysis. The I^2^ value was 71.7% (*P*=0.001) for CHD and 0% (*P*=0.661) for stroke. ALERT indicates Assessment of Lescol in Renal Transplantation Study; CI, confidence interval; CLARICOR, Effect of Clarithromycin on Mortality and Morbidity in Patients With Ischemic Heart Disease; CORONA, Controlled Rosuvastatin Multinational Trial; FINNDIANE, Finnish Diabetic Nephropathy Study; MERLIN‐TIMI36, Metabolic Efficiency With Ranolazine for Less Ischemia in Non–ST‐Elevation Acute Coronary Syndromes Trial; PEACE, Prevention of Events With Angiotensin Converting Enzyme Inhibition Trial; PRACSIS, Prognosis and Risk in Acute Coronary Syndrome in Sweden.

A subset of studies had published risk ratios separately on the secondary outcomes CHD and stroke. When comparing the top versus the bottom tertile of baseline osteoprotegerin concentration, the risk ratio was 1.24 (95% CI, 0.94–1.64; 8 studies; 1592 events; *P*=0.128) for CHD and 1.21 (95% CI, 0.97–1.50; 4 studies; 260 events; *P*=0.090) for stroke (Figure 5). The I^2^ value for between‐study heterogeneity was high for CHD (71.7%; *P*=0.001) and low for stroke (0%; *P*=0.661). Corresponding risk ratios using fixed‐effect meta‐analysis were 1.14 (95% CI, 0.99–1.32; *P*=0.063) for CHD and 1.21 (95% CI, 0.97–1.50; *P*=0.090) for stroke.

### Findings According to Study‐Level Characteristics

**Figure 6:**
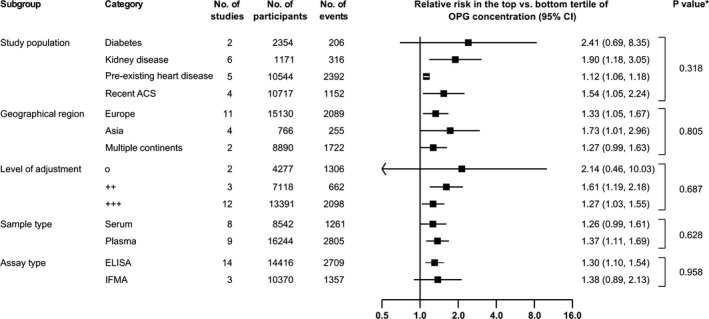
Relative risks for cardiovascular outcomes in individuals in the top vs bottom tertile of osteoprotegerin (OPG) concentration, according to categories of study‐level characteristics. *P values were calculated from meta‐regression. Levels of adjustment: o, unadjusted; ++, adjusted for age, sex, and non–blood‐based risk factors; +++, additionally adjusted for at least 1 blood‐based risk factor. ACS indicates acute coronary syndrome; CI, confidence interval; IFMA, immunofluorometric assay.

Figure 6 presents risk ratios pooled according to study‐level characteristics. There were no significant differences in the strength of association according to population type, geographical region, statistical adjustment, sample type, and assay type (all *P*>0.05). Furthermore, there was no evidence that the strength of association differed according to mean age, sex distribution, or length of follow‐up of the study population (*P* values from meta‐regression: 0.354, 0.170, and 0.564, respectively).

## Discussion

In this literature‐based meta‐analysis, we analyzed 19 high‐risk population studies involving a total of 27 450 participants recruited between 1996 and 2013. Our analysis identified positive associations between osteoprotegerin concentrations and cardiovascular risk. Individuals with a high osteoprotegerin concentration (ie, in the top tertile of baseline osteoprotegerin distribution) had a relative risk of 1.30 (95% CI, 1.12‐1.50) for CVD events when compared with individuals with osteoprotegerin levels in the bottom tertile. This relative risk remained stable under multivariable adjustment and across various study‐level characteristics. The between‐study heterogeneity was high (I^2^=68.3%). Although studies varied in terms of population type, geographical region, level of adjustment, sample type, assay type, proportion of men, mean age, and length of follow‐up, none of these characteristics significantly influenced the strength of association of osteoprotegerin with future CVD risk. However, our analysis identified significant publication bias resulting from predominantly strong positive results in small studies. After correcting for publication bias, the relative risk was reduced to 1.21, but remained significant, with a 95% CI ranging from 1.03 to 1.42. In addition, we confirmed with the leave‐one‐out cross‐validation method that our overall result was not driven by a single study, highlighting the robustness of our finding.

We have previously demonstrated in a literature‐based meta‐analysis that osteoprotegerin is associated with incident CVD in people recruited from the general community.^16^ A combination of findings from 9 general population studies yielded a pooled relative risk for CVD of 1.83 (95% CI, 1.46‐2.30) for a comparison of extreme osteoprotegerin tertiles. In comparison, the present meta‐analysis of studies involving individuals at high CVD risk yielded an association directionally concordant but significantly weaker (Figure S2).

Three distinct features of high‐risk populations may contribute to this weaker association. First, most high‐risk individuals received (multi‐)drug treatment. It has been demonstrated that in vivo treatment with antidiabetic medication,^43^, ^44^, ^45^, ^46^ statins,^47^, ^48^ heparins,^49^, ^50^ or glucocorticoids^51^ and in vitro treatment with irbesartan^52^ or different immunosuppressive therapies^53^ affect circulating osteoprotegerin levels. Second, circulating osteoprotegerin levels differ in people with preexisting diseases. For instance, increased osteoprotegerin levels can be found in patients with preexisting CVD, such as severe peripheral artery disease,^54^ heart failure,^55^ and ST‐segment–elevation acute myocardial infarction.^56^ Moreover, serum osteoprotegerin levels are associated with the presence and severity of coronary artery disease.^57^ Patients with type 1 or type 2 diabetes mellitus exhibit elevated osteoprotegerin levels.^43^, ^44^ High osteoprotegerin values have also been linked to poor glycemic control^58^, ^59^ and severity of diabetic nephropathy.^60^, ^61^ In patients with chronic renal failure, levels of osteoprotegerin are higher compared with healthy controls,^62^ are inversely correlated with glomerular filtration rate,^63^ and correlate with time on maintenance hemodialysis in patients with end‐stage renal disease.^64^ Third, associations of osteoprotegerin may be attenuated because of the dominance of other factors more relevant to CVD risk in high‐risk patients, including highly prevalent traditional CVD risk factors as well as factors related to quality of clinical care, treatment response, or medication adherence.^65^ Altogether, differences in medical treatment, patient histories, disease severity, multimorbidities, and clinical course of disease among high‐risk patients may obscure associations of osteoprotegerin levels with risk for future CVD and might result in reverse causation bias.

The pathophysiological role of osteoprotegerin in CVD development is multifaceted and not completely understood. It is considered to reflect the overall activity of the osteoprotegerin/RANK/RANKL signaling pathway and regulate calcification in both the bone and the vasculature.^9^, ^10^ Osteoprotegerin is expressed in a variety of human tissues^1^; in the vessel wall, it is mainly secreted by endothelial^66^ and vascular smooth muscle cells.^67^ Beneficial effects of osteoprotegerin on the cardiovascular system were reported by several earlier studies. For instance, osteoprotegerin deficiency in mice led to early‐onset osteoporosis and arterial calcification.^68^ Furthermore, osteoprotegerin inactivation in apolipoprotein E–deficient knockout mice increased plaque calcification.^69^ In in vitro studies, osteoprotegerin was found to inhibit calcification in vascular smooth muscle cells^70^ and act as a survival factor in endothelial cells.^71^ In contrast, several lines of evidence from experimental studies in animals and cell cultures suggested harmful effects of osteoprotegerin in agreement with the positive association with CVD risk in our meta‐analysis.^6^ Osteoprotegerin not only contributes to systemic inflammation,^72^ but also to vasculature‐specific inflammation by increasing macrophage infiltration^73^ and leukocyte adhesion to endothelial cells.^74^, ^75^ Moreover, it promotes vascular medial fibrosis^76^ and may exert indirect proatherosclerotic effects by blocking TNF‐related apoptosis‐inducing ligand.^77^ Atherosclerotic plaques that highly express osteoprotegerin exhibit more calcification,^8^ but studies yielded conflicting results about its relevance to plaque stability and conversion to a symptomatic plaque.^52^, ^73^, ^78^, ^79^ These inconsistent reports emphasize the wide‐ranging aspects of osteoprotegerin in the complexity of regulatory processes in atherogenesis and call for more experimental studies to improve our understanding of this pathway in human disease.

Although our meta‐analysis shows positive associations of baseline osteoprotegerin concentration and CVD risk, its incorporation in clinical routine entails some analytical issues. First, available commercial kits for osteoprotegerin measurement use different reference standards of different molecular weight and may, therefore, produce different absolute osteoprotegerin values for the same sample.^80^ Second, previous findings underline the importance of standardized preanalytical and analytical conditions and the need of establishing valid reference ranges for both serum‐ and plasma‐derived blood samples, because osteoprotegerin levels were found lower in serum than in plasma samples.^81^ In analogy to other emerging biomarkers, such as troponin I,^82^ addressing these analytical issues will be an important next step for any use of osteoprotegerin assessment in clinical practice, including the definition of risk thresholds and potential use in risk prediction.

Strengths of the current review include the systematic and comprehensive search of the literature and the standardization of different reported parameters. We rescaled the reported relative risks to reflect a uniform scale (top versus bottom tertile), thereby enabling a direct comparison between the study estimates.^39^ A weakness of the present analysis is that we relied on published information when combining effect estimates from the different studies. A meta‐analysis of individual‐participant data would allow a more consistent approach in defining CVD outcomes and adjusting effect estimates for potential confounding factors. Also, most of the CVD outcomes in our primary analysis were related to CHD and less to stroke. In addition, further investigations on the different components of the osteoprotegerin/RANK/RANKL/TNF‐related apoptosis‐inducing ligand pathway could provide useful pathogenic insight into the role of osteoprotegerin in CVD.

In conclusion, osteoprotegerin is associated with the risk of future CVD in high‐risk populations. The magnitude of association appears weaker than in general population studies.

## Author Contributions

Tschiderer, Klingenschmid, and P. Willeit conducted the systematic literature search, analyzed data, and wrote the article. Nagrani, J. Willeit, Schett, Kiechl, and Laukkanen contributed to writing the discussion and reviewing the article. P. Willeit is the guarantor of this work and, as such, had full access to all the data in the study and takes responsibility for the integrity of the data and the accuracy of the data analysis.

## Sources of Funding

This work was supported by a K‐project grant from the Austrian Research Promotion Agency (“VASCage,” grant 843536).

## Disclosures

None.

## Supporting information


**Table S1.** Search Terms Used to Identify Relevant Articles
**Table S2.** Detailed Baseline Conditions of Contributing Studies
**Figure S1.** Funnel plot including artificial studies generated with the “trim and fill” method.
**Figure S2.** Comparison of combined relative risk for cardiovascular events in the top vs the bottom tertile of osteoprotegerin concentration of high‐risk populations and general population results.^20^
