# Supplementary material for: Osteoprotegerin and Cardiovascular Events in High‐Risk Populations: Meta‐Analysis of 19 Prospective Studies Involving 27 450 Participants
Source: J Am Heart Assoc. 2018 Aug 15;7(16):e009012. doi: 10.1161/JAHA.118.009012 (PMC6201389; doi:10.1161/JAHA.118.009012)
Supplement: Supplementary file 1 — Table S1. Search Terms Used to Identify Relevant Articles Table S2. Detailed Baseline Conditions of Contributing Studies Figure S1. Funnel plot including artificial studies generated with the “trim and fill” method. Figure S2. Comparison of combined relative risk for cardiovascular events in the top vs the bottom tertile of osteoprotegerin concentration of high‐risk populations and general population results.20 [file JAH3-7-e009012-s001.pdf]

SUPPLEMENTAL MATERIAL

Table S1. Search terms used to identify relevant articles
**PubMed**
("OPG" [All Fields] OR "Osteoprotegerin" [All Fields] OR "OCIF Protein" [All Fields] OR "Osteoclastogenesis Inhibitory Factor" [All Fields] OR "Tumor Necrosis Factor Receptor 11b" [All Fields] OR "Osteoprotegerin"[MeSH]) AND ("Cardiovascular Diseases" [MeSH] OR "Coronary Artery Disease" [MeSH] OR "Atherosclerosis" [MeSH] OR "Coronary Disease" [MeSH] OR "Myocardial Infarction" [MeSH] OR "Myocardial Ischemia" [MeSH] OR "Stroke" [MeSH] OR "Cerebrovascular" [All fields]) NOT ("Animals"[MeSH] NOT "Humans"[MeSH])
**Web of Science**
TS=("OPG" OR "Osteoprotegerin" OR "OCIF Protein" OR "Osteoclastogenesis Inhibitory Factor" OR "Tumor Necrosis Factor Receptor 11b") AND TS=("Cardiovascular Diseases" OR "Coronary Artery Disease" OR "Atherosclerosis" OR "Coronary Disease" OR "Myocardial Infarction" OR "Myocardial Ischemia" OR "Stroke" OR "Cerebrovascular")
**EMBASE**
("OPG" OR "Osteoprotegerin" OR "OCIF Protein" OR "Osteoclastogenesis Inhibitory Factor" OR "Tumor Necrosis Factor Receptor 11b").af AND ("Cardiovascular Diseases" OR "Coronary Artery Disease" OR "Atherosclerosis" OR "Coronary Disease" OR "Myocardial Infarction" OR "Myocardial Ischemia" OR "Stroke" OR "Cerebrovascular").af

No language restrictions were applied.

Table S2. Detailed baseline conditions of contributing studies	
**Study acronym or first author**	**Baseline diseases**
**Populations with diabetes at baseline**	
Anand[1]	Type 2 diabetes
FINNDIANE[2]	Type 1 diabetes
**Populations with kidney disease at baseline**	
ALERT[3]	Renal transplant recipients (did not report on cause of kidney disease)
CRISIS[4]	All cause chronic kidney disease
Kuzniewski[5]	Chronic glomerulonephritis, pyelonephritis, polycystic kidney disease, diabetic nephropathy, unknown cause
Nakashima[6]	Long term hemodialysis patients (did not report on cause of kidney disease)
Nishiura[7]	Consecutive hemodialysis patients (diabetic nephropathy, chronic glomerulonephritis, nephrosclerosis, other cause)
Speer[8]	Chronic hemodialysis patients (vascular-tubulointerstitial [including hypertension], diabetes mellitus, glomerulonephritis, other cause)
Yilmaz[9]	Chronic kidney disease (did not report on cause of kidney disease)
**Populations with pre-existing heart disease at baseline**	
CLARICOR[10]	Stable coronary artery disease
CORONA[11]	Chronic heart failure of ischemic cause (NYHA class II to IV, LV ejection fraction ≤40% [≤35% if NYHA II])
Jono[12]	Stable coronary artery disease
PEACE[13]	Stable coronary artery disease

Pedersen (1)[14]	Stable angina pectoris
**Populations with recent acute coronary syndromes at baseline**	
MERLIN-TIMI36[15]	Non-ST elevation acute coronary syndromes
OPTIMAAL[16]	Acute myocardial infarction complicated with heart failure during the acute phase
PLATO[17]	Acute coronary syndromes
PRACSIS[18]	Acute coronary syndromes (unstable angina, non-ST-segment elevation myocardial infarction, or ST-segment elevation myocardial infarction)
Pedersen (2)[19]	ST-segment elevation myocardial infarction treated with primary percutaneous coronary intervention

ALERT, Assessment of Lescol in Renal Transplantation Study; CLARICOR, Effect of Clarithromycin on Mortality and Morbidity in Patients With Ischemic Heart Disease; CORONA, Controlled Rosuvastatin Multinational Trial; CRISIS, Chronic Renal Insufficiency Standards Implementation Study; FINNDIANE, Finnish Diabetic Nephropathy Study; MERLIN-TIMI36, Metabolic Efficiency with Ranolazine for Less Ischemia in Non–ST-elevation acute coronary syndromes trial; NYHA, New York Heart Association; OPTIMAAL, Optimal Trial in Myocardial Infarction with Angiotensin II Antagonist Losartan; PEACE, Prevention of Events with Angiotensin Converting Enzyme Inhibition Trial; PLATO, PLATelet inhibition and patient Outcomes trial; PRACSIS, Prognosis and Risk in Acute Coronary Syndrome in Sweden.

Figure S1. Funnel plot including artificial studies generated with the ‘trim and fill’ method.

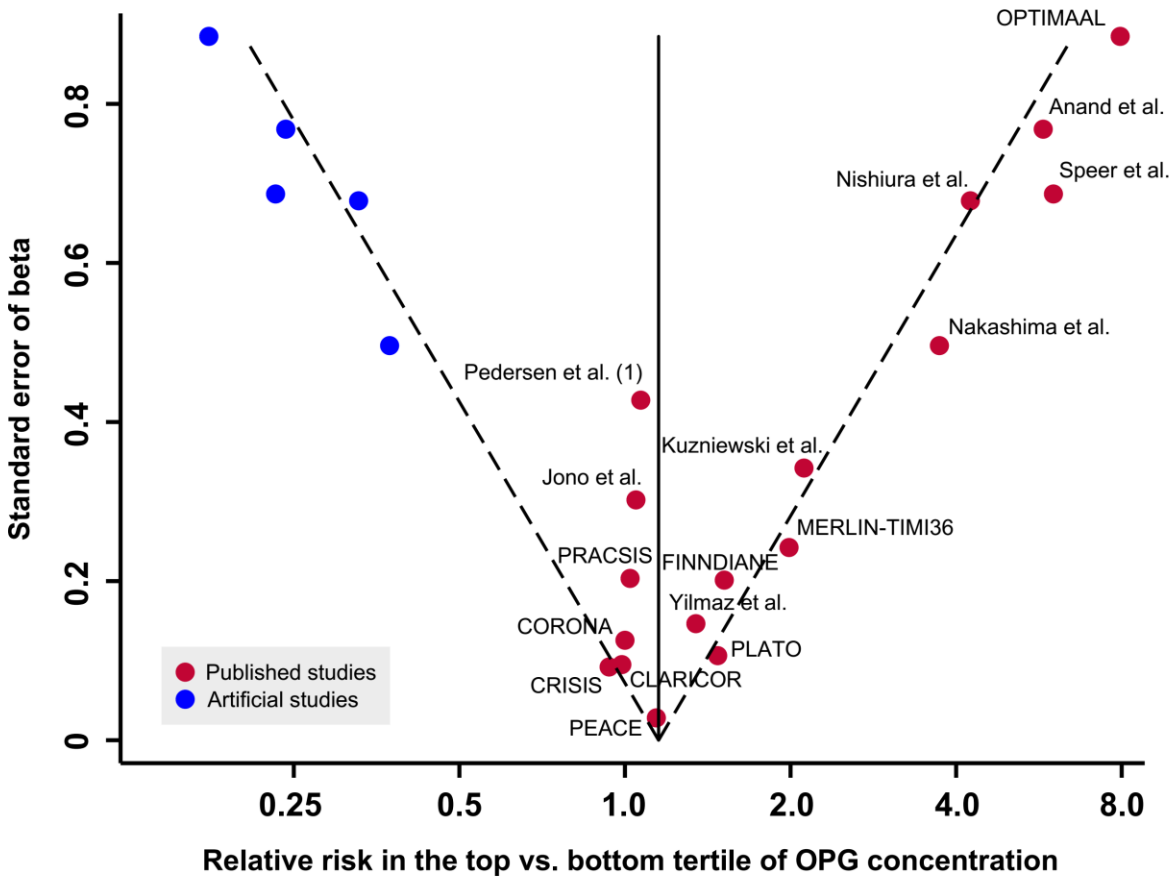

Full study names are listed in the footnote of **Table S2**. The dotted lines show pseudo 95% confidence intervals around the overall pooled estimate. The P value from Egger’s asymmetry test of associations was 0.013. Blue dots indicate artificial studies included by the ‘trim and fill’ method. OPG, osteoprotegerin.

Figure S2. Comparison of combined relative risk for cardiovascular events in the top vs. the bottom tertile of osteoprotegerin concentration of high-risk populations and general population results[20].

Study	No. of participants	No. of events	Relative risk in the top vs. bottom tertile of OPG concentration (95% CI)	% weight
**General population**				
Browner et al.	490	51	1.96 (1.01, 3.80)	7.65
BRUN	909	124	1.80 (1.04, 3.11)	9.70
FRAM-OC	2933	132	1.92 (1.25, 2.97)	12.29
Shen et al.	6092	118	2.26 (1.51, 3.39)	13.00
CAIFOS	1292	210	1.30 (1.00, 1.68)	17.11
Tromso	5699	909	1.56 (1.32, 1.85)	19.65
CCHS	5863	616	2.39 (2.10, 2.73)	20.60
**Subtotal**	**23278**	**2160**	**1.83 (1.46, 2.30)**	**100.00**
**High-risk population**				
OPTIMAAL	234	26	7.95 (1.40, 45.04)	0.66
Anand et al.	510	16	5.76 (1.28, 25.96)	0.86
Speer et al.	98	23	6.01 (1.56, 23.09)	1.06
Nishiura et al.	99	27	4.25 (1.12, 16.04)	1.08
Nakashima et al.	151	40	3.73 (1.41, 9.86)	1.89
Pedersen et al. (1)	1025	60	1.07 (0.46, 2.47)	2.43
Kuzniewski et al.	69	31	2.12 (1.08, 4.14)	3.45
Jono et al.	225	101	1.05 (0.58, 1.89)	4.14
MERLIN-TIMI36	4463	544	1.99 (1.24, 3.20)	5.51
PRACSIS	897	150	1.02 (0.69, 1.52)	6.70
FINNDIANE	1903	190	1.52 (1.02, 2.25)	6.77
Yilmaz et al.	291	87	1.35 (1.01, 1.79)	8.90
CORONA	1464	318	1.00 (0.78, 1.28)	9.82
PLATO	5123	432	1.47 (1.20, 1.82)	10.69
CLARICOR	4063	623	0.99 (0.82, 1.19)	11.20
CRISIS	463	108	0.94 (0.78, 1.12)	11.33
PEACE	3767	1290	1.14 (1.08, 1.20)	13.51
**Subtotal**	**24845**	**4066**	**1.30 (1.12, 1.50)**	**100.00**
**Overall**	**48123**	**6226**	**1.54 (1.31, 1.81)**	

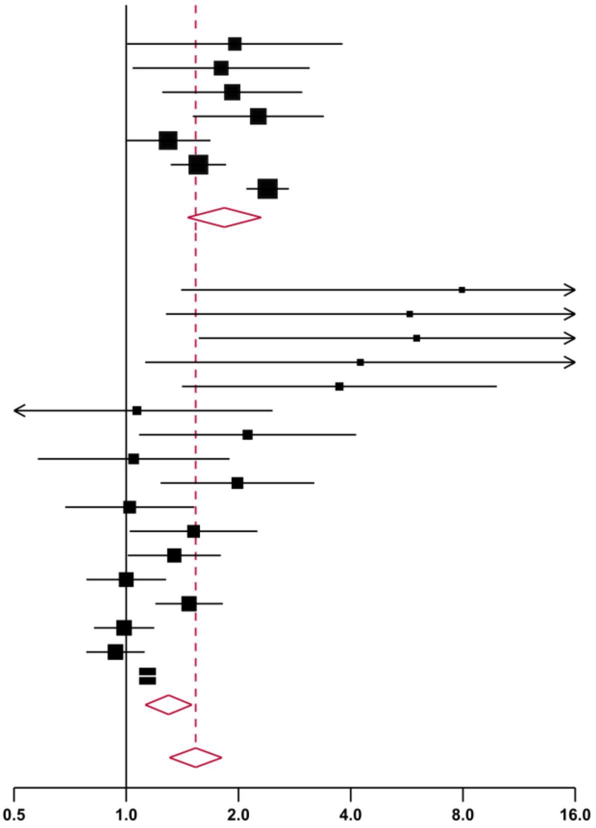

Sizes of data markers indicate the weight of each study in the analysis. Study-specific relative risks were pooled using random-effects meta-analysis. Full names of studies in high-risk populations are listed in the footnote of **Table S2**. The overall I^2 value was 87.0% (P<0.001). BRUN, Bruneck Study; CAIFOS, Calcium Intake Fracture Outcome study; CCHS, Copenhagen City Heart Study; CI, confidence interval; FRAM-OC, Framingham Study-Offspring Cohort; OPG, osteoprotegerin; Tromso, The Tromso Study.

Supplemental References:

1. Anand DV, Lahiri A, Lim E, Hopkins D, Corder R. The relationship between plasma osteoprotegerin levels and coronary artery calcification in uncomplicated type 2 diabetic subjects. *J Am Coll Cardiol*. 2006;47:1850–1857.
2. Gordin D, Soro-Paavonen A, Thomas MC, Harjutsalo V, Saraheimo M, Bjerre M, Forsblom C, Flyvbjerg A, Groop P-H. Osteoprotegerin is an independent predictor of vascular events in Finnish adults with type 1 diabetes. *Diabetes Care*. 2013;36:1827–1833.
3. Svensson M, Dahle DO, Mjoen G, Weihrauch G, Scharnagl H, Dobnig H, Marz W, Jardine A, Fellstrom B, Holdaas H. Osteoprotegerin as a predictor of renal and cardiovascular outcomes in renal transplant recipients: follow-up data from the ALERT study. *Nephrol Dial Transplant*. 2012;27:2571–2575.
4. Alderson HV, Ritchie JP, Middleton R, Larsson A, Larsson TE, Kalra PA. FGF-23 and Osteoprotegerin but not Fetuin-A are associated with death and enhance risk prediction in non-dialysis chronic kidney disease stages 3-5. *Nephrology (Carlton)*. 2016;21:566–573.
5. Kuzniewski M, Fedak D, Dumnicka P, Stepien E, Kusnierz-Cabala B, Cwynar M, Sulowicz W. Osteoprotegerin and osteoprotegerin/TRAIL ratio are associated with cardiovascular dysfunction and mortality among patients with renal failure. *Adv Med Sci*. 2016;61:269–275.
6. Nakashima A, Carrero JJ, Qureshi AR, Hirai T, Takasugi N, Ueno T, Taniguchi Y, Lindholm B, Yorioka N. Plasma osteoprotegerin, arterial stiffness, and mortality in normoalbuminemic Japanese hemodialysis patients. *Osteoporos Int*. 2011;22:1695–1701.
7. Nishiura R, Fujimoto S, Sato Y, Yamada K, Hisanaga S, Hara S, Nakao H, Kitamura K. Elevated osteoprotegerin levels predict cardiovascular events in new hemodialysis patients. *Am J Nephrol*. 2009;29:257–263.

8. Speer G, Fekete BC, El Hadj Othmane T, Szabo T, Egresits J, Fodor E, Kiss I, Logan AG, Nemcsik J, Szabo A, Nemeth ZK, Szathmari M, Tisler A. Serum osteoprotegerin level, carotid-femoral pulse wave velocity and cardiovascular survival in haemodialysis patients. *Nephrol Dial Transplant*. 2008;23:3256–3262.
9. Yilmaz MI, Siriopol D, Saglam M, Unal HU, Karaman M, Gezer M, Kilinc A, Eyileten T, Guler AK, Aydin I, Vural A, Oguz Y, Covic A, Ortiz A, Kanbay M. Osteoprotegerin in Chronic Kidney Disease: Associations with Vascular Damage and Cardiovascular Events. *Calcif Tissue Int*. 2016;99:121–130.
10. Bjerre M, Hilden J, Kastrup J, Skoog M, Hansen JF, Kolmos HJ, Jensen GB, Kjoller E, Winkel P, Flyvbjerg A, Gluud C. Osteoprotegerin independently predicts mortality in patients with stable coronary artery disease: the CLARICOR trial. *Scand J Clin Lab Invest*. 2014;74:657–664.
11. Ueland T, Dahl CP, Kjekshus J, Hulthe J, Bohm M, Mach F, Goudev A, Lindberg M, Wikstrand J, Aukrust P, Gullestad L. Osteoprotegerin predicts progression of chronic heart failure: results from CORONA. *Circ Heart Fail*. 2011;4:145–152.
12. Jono S, Otsuki S, Higashikuni Y, Shioi A, Mori K, Hara K, Hashimoto H, Ikari Y. Serum osteoprotegerin levels and long-term prognosis in subjects with stable coronary artery disease. *J Thromb Haemost*. 2010;8:1170–1175.
13. Omland T, Cristophi C, Flyvbjerg A, Rice MM, Jablonski KA, Rasmussen L, Rouleau JL, Sabatine MS, Pfeffer MA, Braunwald E. Abstract P653: Circulating osteoprotegerin levels are associated with peripheral vascular intervention and stroke in patients with stable coronary artery disease: the PEACE trial. *Eur Heart J*. 2008;29:85.
14. Pedersen ER, Ueland T, Seifert R, Aukrust P, Schartum-Hansen H, Ebbing M, Bleie O, Igland J, Svingen G, Nordrehaug JE, Nygard O. Serum osteoprotegerin levels and long-term prognosis in patients with stable angina pectoris. *Atherosclerosis*. 2010;212:644–649.

15. Roysland R, Bonaca MP, Omland T, Sabatine M, Murphy SA, Scirica BM, Bjerre M, Flyvbjerg A, Braunwald E, Morrow DA. Osteoprotegerin and cardiovascular mortality in patients with non-ST elevation acute coronary syndromes. *Heart*. 2012;98:786–791.
16. Ueland T, Jemtland R, Godang K, Kjekshus J, Hognestad A, Omland T, Squire IB, Gullestad L, Bollerslev J, Dickstein K, Aukrust P. Prognostic value of osteoprotegerin in heart failure after acute myocardial infarction. *J Am Coll Cardiol*. 2004;44:1970–1976.
17. Ueland T, Åkerblom A, Ghukasyan T, Michelsen AE, Aukrust P, Becker RC, Bertilsson M, Himmelmann A, James SK, Siegbahn A, Storey RF, Kontny F, Wallentin L. Abstract 12851: Osteoprotegerin is Associated With Major Bleeding but Not With Cardiovascular Outcomes in Patients With Acute Coronary Syndromes - Insights From the PLATelet Inhibition and Patient Outcomes (PLATO) Trial. *Circulation*. 2016;134:A12851.
18. Omland T, Ueland T, Jansson AM, Persson A, Karlsson T, Smith C, Herlitz J, Aukrust P, Hartford M, Caidahl K. Circulating osteoprotegerin levels and long-term prognosis in patients with acute coronary syndromes. *J Am Coll Cardiol*. 2008;51:627–633.
19. Pedersen S, Mogelvang R, Bjerre M, Frystyk J, Flyvbjerg A, Galatius S, Sorensen TB, Iversen A, Hvelplund A, Jensen JS. Osteoprotegerin predicts long-term outcome in patients with ST-segment elevation myocardial infarction treated with primary percutaneous coronary intervention. *Cardiology*. 2012;123:31–38.
20. Tschiderer L, Willeit J, Schett G, Kiechl S, Willeit P. Osteoprotegerin concentration and risk of cardiovascular outcomes in nine general population studies: Literature-based meta-analysis involving 26,442 participants. *PLoS One*. 2017;12:e0183910. doi: 10.1371/journal.pone.0183910.
